# Supplementary figures and images for: Resistin production does not affect outcomes in a mouse model of acute surgical sepsis
Source: PLoS One. 2022 Mar 14;17(3):e0265241. doi: 10.1371/journal.pone.0265241 (PMC8920279; doi:10.1371/journal.pone.0265241)

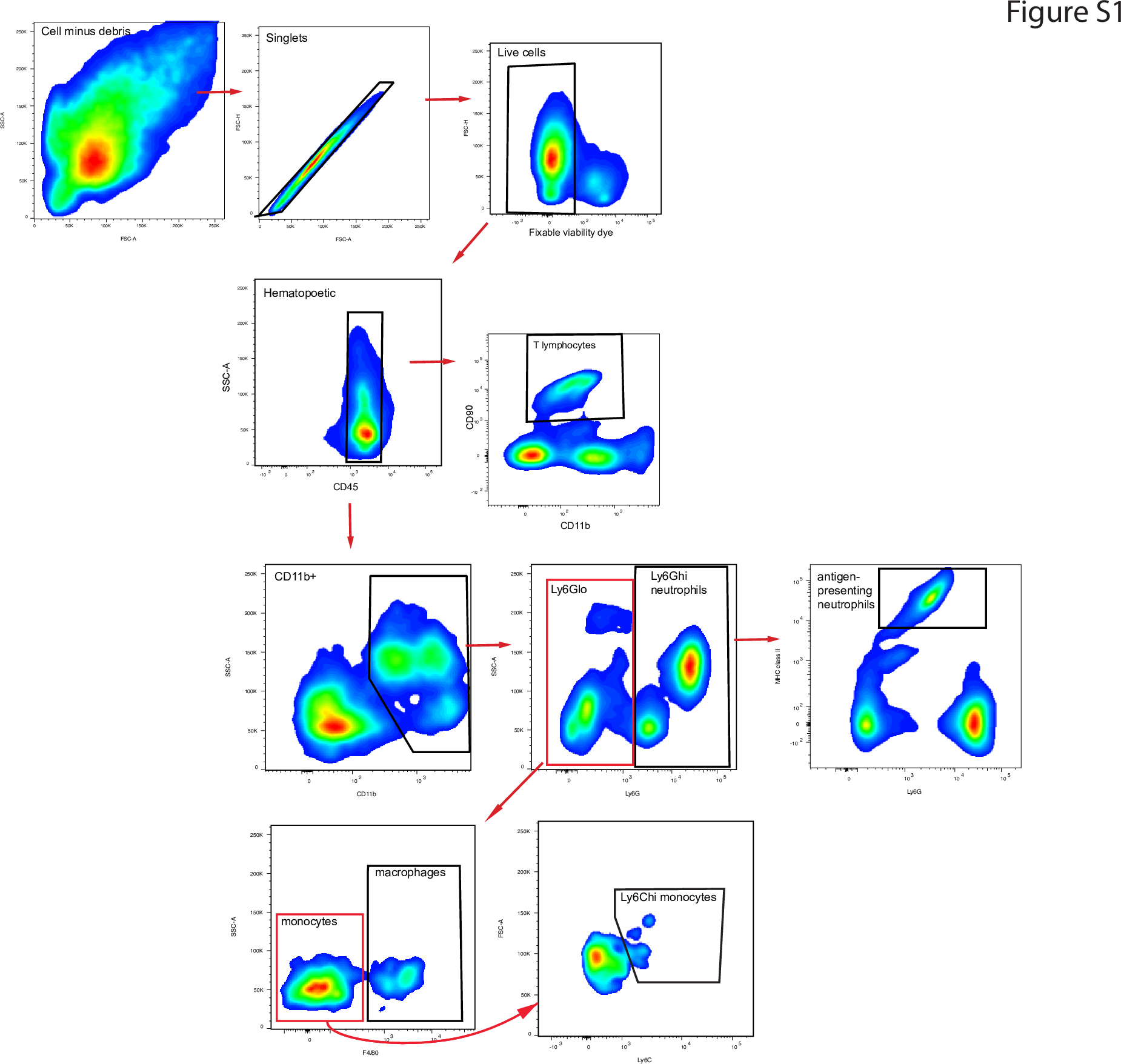

Supplement: S1 Fig — Representative flow cytometry profiling for peritoneal fluid analysis obtained 24 h after acute sepsis. (TIF) [file pone.0265241.s001.tif]

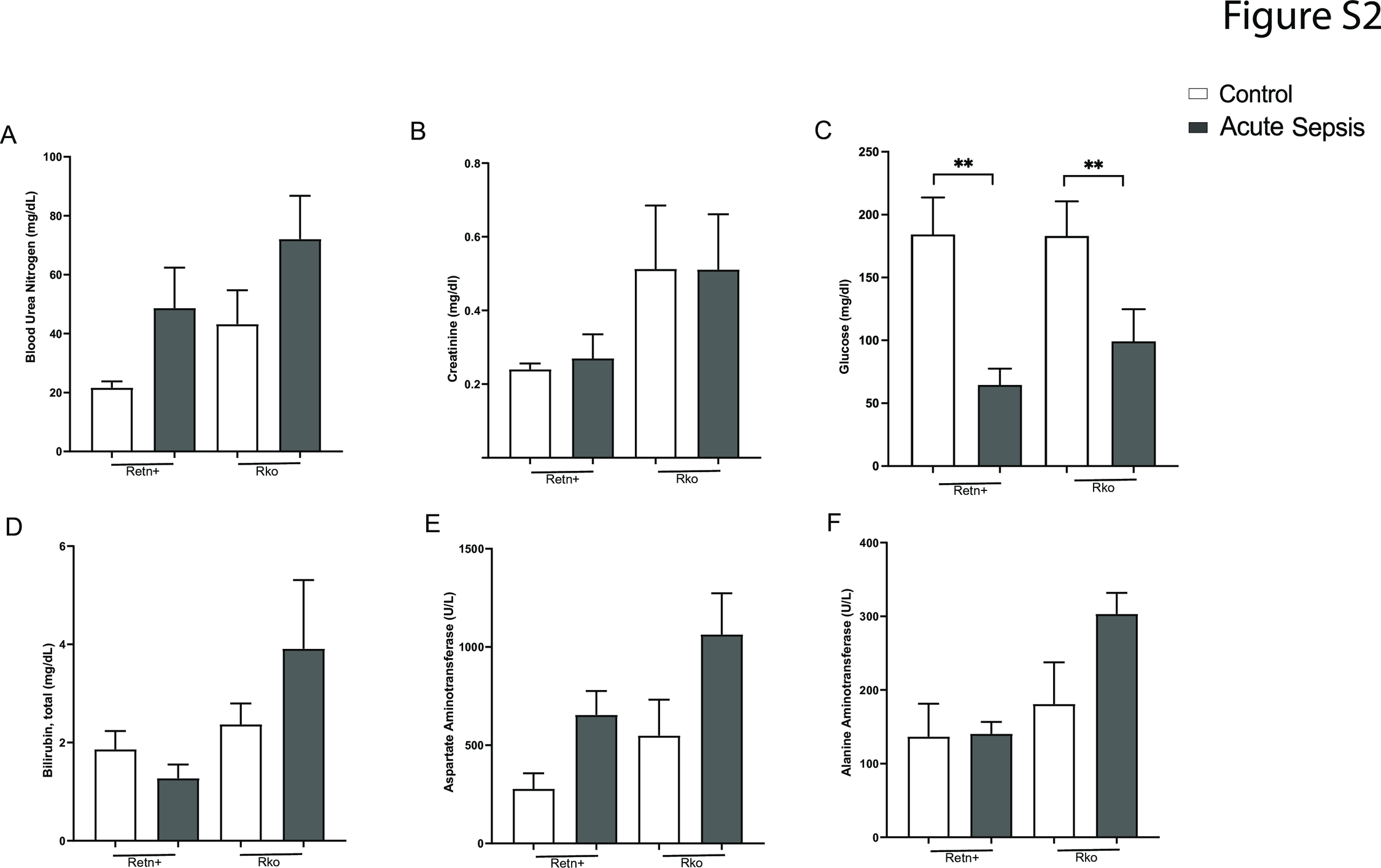

Supplement: S2 Fig — A—F represent mean (+/- SEM) serum concentrations of the named biomarkers at 24 h following surgery, of which only glucose concentration varies among control and acute sepsis (p = 0.009 for Retn+ mice; p = 0.005 for Rko mice). n = 9–13 per group. (TIF) [file pone.0265241.s002.tif]

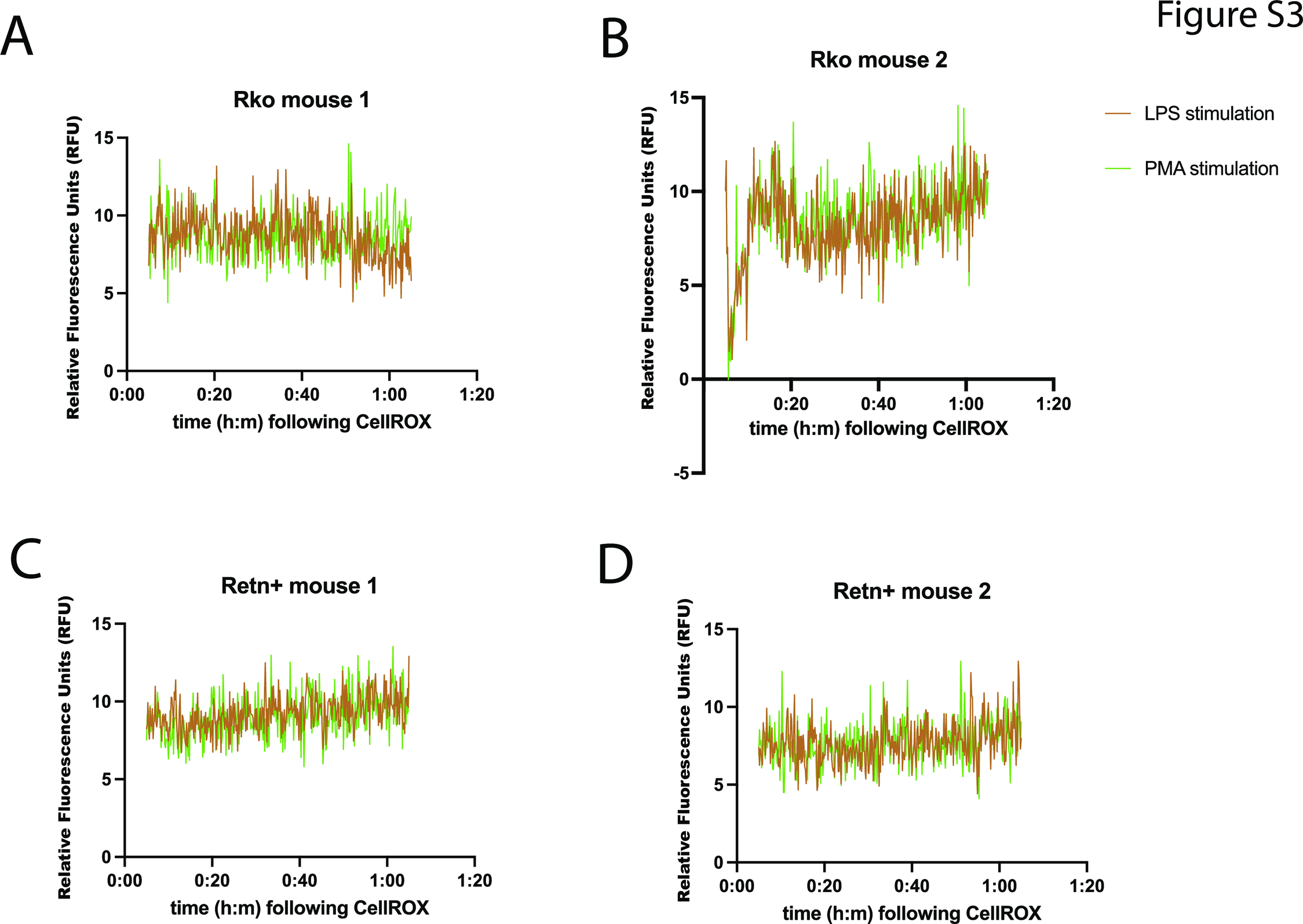

Supplement: S3 Fig — Kinetics of CellROX Deep Red Reagent, from 5 min to 65 min following the addition of CellROX reagent to neutrophils isolated from bone marrow of Rko (A, B) or Retn+ (C, D) mice. Values represent average fluorescence emission (minus control fluorescence) from 1.5 million cells primed with 100ng/ml PMA or LPS for 2 h at 38°C. (TIF) [file pone.0265241.s003.tif]
