# Supplementary material for: Resistin production does not affect outcomes in a mouse model of acute surgical sepsis
Source: PLoS One. 2022 Mar 14;17(3):e0265241. doi: 10.1371/journal.pone.0265241 (PMC8920279; doi:10.1371/journal.pone.0265241)
Supplement: S1 File — (DOCX) [file pone.0265241.s004.docx]

**Supporting Material**

**Table S1:** Mean (+/- SEM) blood cytokine concentrations, as measured by Multiplex Analysis, at 6 h following acute sepsis in Retn+ and Rko mice. Mixed-effects (Šídák) analysis of sepsis effect versus genotype in these analytes was not statistically significant.

|  | **Rko Control** | **Rko Acute Sepsis** | **Retn+ Control** | **Retn+ Acute Sepsis** |  |
| --- | --- | --- | --- | --- | --- |
|  |  |  |  |  |  |
| **SP-D** | 4259.7 (1189.2) | 4829.6 (71.3) | 3170.1 (268.6) | 19900.1 (15202.5) |  |
| **TNF-alpha** | 5.9 (4.6) | 4.6 (0.3) | 8.74 (7.53) | 10.5 (4.6) |  |
| **IL-12 p70** | 68.2 (14.1) | 78.4 (10.1) | 24.6 (15.9) | 69.8 (35.7) |  |
| **IL-10** | 301.7 (294.1) | 60.3 (27.3) | 345.7 (335.3) | 243.8 (136.3) |  |
| **P-selectin/CD62P** | 38107.5 (2699) | 46630.6 (8429.6) | 50891.1 (3010.9) | 44390.5 (7991.8) |  |
| **syndecan-1/CD138** | 9115.0 (1841.0) | 9072.3 (2742.9) | 7676.9 (388.7) | 9286.3 (1133.9) |  |
| **IL-27** | 18.4 (11.6) | 17.4 (3.3) | 8.52 (2.34) | 21.9 (7.6) |  |
| **uPAR** | 2551.4 (527.5) | 2932.4 (301.7) | 2154.0 (412.8) | 3672.4 (972.4) |  |
| **RAGE/AGER** | 42.9 (16.0) | 67.7 (8.2) | 23.3 (11.3) | 778.5 (759.0) |  |

**Table S2:** Mean (+/- SEM) blood concentrations, as measured by Multiplex Analysis, at 24 h following acute sepsis in Retn+ and Rko mice. Mixed-effects (Šídák) analysis of sepsis effect versus genotype versus time (matched analytes compared between 6 h and 24 h) were not statistically significant.

|  | **Rko Control** | **Rko Acute Sepsis** | **Retn+ Control** | **Retn+ Acute Sepsis** |  |
| --- | --- | --- | --- | --- | --- |
|  |  |  |  |  |  |
| **SP-D** | 3138.7 (216.0) | 70487.51 (46826.63) | 3964.4 (966.1) | 17487.98 (5775.00) |  |
| **TNF-alpha** | 1.21 (0.1) | 3.45 (0.16) | 6.2 (5.1) | 4.84 (2.08) |  |
| **IL-12 p70** | 33.2 (11.9) | 49.37 (3.81) | 235.6 (10.0) | 44.27 (5.72) |  |
| **IL-10** | 7.8 (1.2) | 125.92 (30.63) | 10709.9 (1063.5) | 245.04 (146.20) |  |
| **P-selectin/CD62P** | 32728.3 (2771.5) | 43380.82 (6985.95) | 33706.8 (4726.6) | 50286.05 (15331.67) |  |
| **syndecan-1/CD138** | 5684.6 (400.6) | 12646.09 (1182.44) | 11643.0 (5336.7) | 21891.62 (4841.85) |  |
| **IL-27** | 4.35 (0.9) | 8.99 (0.80) | 10.7 (6.0) | 8.44 (2.46) |  |
| **uPAR** | 1585.8 (96.9) | 2798.7 (173.20) | 2511.6 (859.0) | 2877.74 (698.17) |  |
| **RAGE/AGER** | 20.7 (8.57) | 40.73 (19.53) | 16.4 (5.5) | 30.75 (13.28) |  |
